# Exposure to Literary Fiction Is Associated With Lower Psychological Essentialism

**DOI:** 10.3389/fpsyg.2021.662940

**Published:** 2021-06-08

**Authors:** Emanuele Castano, Maria Paola Paladino, Olivia G. Cadwell, Valentina Cuccio, Pietro Perconti

**Affiliations:** ^1^Department of Psychology and Cognitive Science, University of Trento, Trento, Italy; ^2^Department of Psychology, The New School, New York City, NY, United States; ^3^COSPECS, University of Messina, Messina, Italy

**Keywords:** literary fiction, linguistic inferences, theory of mind, social cognition, psychological essentialism, fiction (narrative), cognitive literary theory

## Abstract

We investigated the impact of exposure to literary and popular fiction on psychological essentialism. Exposure to fiction was measured by using the Author Recognition Test, which allows us to separate exposure to authors of literary and popular fiction. Psychological essentialism was assessed by the discreteness subscale of the psychological essentialism scale in Study 1, and by the three subscales of the same scale (such as discreteness, informativeness, and biological basis) in Study 2 that was pre-registered. Results showed that exposure to literary fiction negatively predicts the three subscales. The results emerged controlling for political ideology, a variable that is commonly associated with psychological essentialism, and level of education.

## Introduction

Oscar Wilde suggested, or more likely stated, that “You are what you read.” This sweeping statement is hardly disprovable, but various aspects of it have been applied for the test through empirical studies in a variety of disciplines ranging from linguistics to developmental psychology, and from literary studies to cognitive science. Reading affects both *what* we learn and *how* we learn about the world (Heyes, 2012).

One of the most fascinating aspects of reading is that when reading, we go well-beyond what is literally said. Guided by pragmatic competence, we fill the gaps chiefly through the process of inference (Grice, 1957; Recanati, 2004). Inference-making occurs when we read all kinds of texts, but it is perhaps at its fullest when we engage with narrative texts (Bruner, 1986), the comprehension of which is aided by the creation and integration of mental representations of the characters, events, and context of the story (Graesser et al., 1991; Zwaan et al., 1995; Zwaan and Radvansky, 1998; Zwaan, 1999, 2004). In human development, the comprehension of narrative texts appears earlier than the comprehension of other expository text and other genres (Kaplan, 2013), which is consistent with the fact that stories played a significant role in the evolution of *Homo sapiens* well before the practice of writing and reading (e.g., Boyd, 2010). According to anthropologist Polly Wiessner, stories played such a role by “evoking higher orders of theory of mind *via* the imagination, conveying attributes of people in broad networks (virtual communities), and transmitting the ‘big picture' of cultural institutions that generate regularity of behavior, cooperation, and trust at the regional level” (Wiessner, 2014, p. 1).

The theoretical and empirical findings that have emerged over the last two decades support the conclusion of the study by Wiessner, particularly with regard to the relationship between engagement with narrative fiction and the development of Theory of Mind (ToM; Dodell-Feder and Tamir, 2018). This article is based on this line of inquiry. Building on the existent theory based on the difference between literary and popular fiction, and on research showing their differential impact on social cognition (Kidd and Castano, 2013), we investigated whether engagement with literary, but not popular fiction, reduces psychological essentialism.

## Narrative Fiction and Theory of Mind

The capacity of narrative fiction to transport the reader into the mind of characters has long been noted, but only in the work of cognitive literary theorist Lisa Zunshine this phenomenon has been thoroughly described. The traditional literary critical analysis of the practice of reading and writing describes this ability in terms of *imagination* and *pretense*. Zunshine emphasized, and convincingly showed, that our ability to make sense of fiction relies heavily on cognitive processes such as mind-reading and meta-representationality (Zunshine, 2006). This work in the tradition of literary studies is similar to psychological research.

Building on early insight demonstrating that when we read narrative fiction, we experience thoughts and emotions congruent with that of the fictional characters (Gerrig, 1993; Oatley, 1999), and researchers began exploring the relationship between reading fiction and ToM (Premack and Woodruff, 1978). Also known as taking an intentional stance (Dennett, 1989), or mentalizing (Frith, 1989), ToM, in its advanced form, can be defined as the capacity to infer and represent the mental states of other people. Is ToM enhanced by fiction reading? Mar et al. measured exposure to fiction by using an adapted version of the Author Recognition Test (ART) suggested by Stanovich and West (1989), which consisted of names of authors of narrative fiction and authors of non-fiction, and asked participants to select the name of authors that they recognized. This allowed the computation of exposure to fiction and non-fiction. Subsequently, participants performed a series of tests, including the Reading the Mind in the Eyes Test (RMET; Baron-Cohen et al., 2001). The RMET, among the most widely used measures of ToM, is a performance measure, which assesses the accuracy in inferring the mental states from the photographs of the eye region. Results showed that exposure to narrative fiction, but not non-fiction, is positively related to ToM (Mar et al., 2006).

The results of these studies are interpreted as stemming from the fact that narrative fiction simulates social life: readers are transported into the fictional world, and they identify with characters, feel their emotions, and imagine their thoughts and desires. In doing so, they engage in mentalizing and thus train their ToM skills (Oatley, 2016). Recent research has qualified this conclusion and proposed a complementary account of the effect of reading fiction on mind-reading.

In a series of experiments, participants were first randomly assigned to read one among many excerpts of novels or short stories that were categorized either as literary or as popular fiction, and they were then asked to complete the RMET. Results showed that participants in the literary fiction condition scored higher on the test compared with those in the popular fiction condition. The literary fiction condition participants also scored higher than those who were either in a non-fiction reading condition or in a condition in which they did not read anything at all (Kidd and Castano, 2013; see also Black and Barnes, 2015; Kidd et al., 2016; Pino and Mazza, 2016; Kidd and Castano, 2018; van Kuijk et al., 2018; but see Panero et al., 2016; Kidd and Castano, 2017, 2018; for a meta-analysis, see Dodell-Feder and Tamir, 2018).

The differential impact of exposure to literary vs. popular fiction is further supported by the research using the ART, the same instrument originally used by Mar et al. (2006) when exploring the relation between fiction reading and ToM. When Kidd and Castano (2017) factor-analyzed the answers to the version of the ART suggested by Acheson et al. (2008), which are provided by a large number of participants, they identified two factors that correspond to the recognition of literary vs. popular genre authors (see also Moore and Gordon, 2015). The literary fiction factor comprises authors such as Michael Ondaatje, Thomas Pynchon, Margaret Atwood, and Alice Walker. The popular fiction factor comprises authors such as Tom Clancy, Nelson DeMille, Danielle Steel, and James Patterson (for a complete list of authors and their loadings on the two factors, see Kidd and Castano, 2017). After assigning a separate score representing familiarity with literary fiction and familiarity with popular fiction to each participant, they used both of these scores in a multiple regression analysis with performance on the RMET as a criterion. Resembling these experimental findings, only exposure to literary fiction emerged as a predictor and this relation was not accounted for by the differences in gender, age, undergraduate major, level of education, or self-reported trait empathy. Kidd and Castano (2017) also computed a variety of checks with regard to literary and popular factors of the ART, notably checking for the number of authors, the publication date of the work by the authors, specific genres, and the presence of classic authors. These checks did not alter the findings, and the two factors, namely, literary, and popular, were also used in the research that followed, in which they differentially predicted thinking styles and social cognition biases (Castano et al., 2020). Thus, it appears that, notwithstanding the imperfection of the ART as a measure of exposure to fiction, the instrument can be considered a valid proxy for exposure to fiction and computing the two separate scores is meaningful and statistically significant (Kidd and Castano, 2017). The ART is further discussed in the following section.

## Narrative Fiction and Social Categories

The rationale behind the studies reviewed earlier is that literary fiction revolves around complex, *round* (Forster, 2002) characters, whose mental life is not explicitly revealed. This requires a mentalizing effort, which results in priming and training of ToM processes. The simpler, *flat* (Forster, 2002) characters of popular fiction do not require the same mentalizing effort. Recent experimental research confirms that characters of literary fiction are perceived as more complex than those of popular fiction (Kidd and Castano, 2019).

Another way to consider the differences between popular and literary fiction is how these differences map onto the distinction made in social cognition research between the category-based and individuated perceptions (Brewer, 1988; Fiske and Neuberg, 1990). In his analysis of characterization in fiction, Culpeper (2001) proposed that the flat characters of popular fiction are easily recognized as fitting certain social categories (adolescent or elderly, Latino or WASP; Tajfel, 1981) a social role (Goffman, 1956), or, we suggested, as exemplars of a certain personality type (such as the extroverted type and the neurotic type) (McCrae and Costa, 1999). The characters of literary fiction, on the other hand, are category-resistant; these characters do not easily fit the social categories that we routinely use to make sense of the social world (Eder et al., 2010; Keen, 2011; Kidd and Castano, 2017), and those characters are thus more likely to be appraised through individuated perception. From the social cognition research, we also learn that we appraise others in a person-based, individuated manner only when we are motivated to do so (e.g., because we are in a competitive interpersonal relation; Ruscher and Fiske, 1990) or when the information about the person challenges the group stereotype (Rubinstein et al., 2018).

In a novel by, say, Tony Hillerman or James Patterson, the category-based appraisal may work, and the schema for the appropriate social categories might even be reinforced. In contrast, if we are engaging with the characters in a novel by Thomas Pynchon or Virginia Woolf, because characters are more complex and resist social categorization, individuated appraisal is more likely. Being confronted with these literary fiction characters may, in the long run, diminish our propensity to appraise the world in terms of social categories. If people keep encountering, either in real life or in the fictional world of novels, who are overly emotional British (or unemotional Italians), emotionally attuned engineers, and empathic bankers, then they may come to perceive British (and Italians), engineers, and bankers categories as loose associations, rather than discrete, entitative categories (Castano et al., 2002; Yzerbyt et al., 2004). Similarly, if the behavior of a fictional character suggests extraversion in one context but introversion in another, the belief that people are either extroverted or introverted may be undermined.

Since characters of literary fiction undermine the reification of social categories and of social types, such as personality types, exposure to literary fiction should reduce psychological essentialism.

## Psychological Essentialism

Being an essentialist means believing that behind the variety of observable phenomena, there are essences that explain their systematic nature; essentialism means belief in the metaphysical claim that there are natural essences that explain the surface similarity. This stance is rooted in the history of thought, at least since the time of Plato and Aristotle, and is still very much alive in contemporary thought (e.g., Putnam, 1975). How do we speak of “cats” given the manifold manifestations of this phenomenon in our ordinary experience? The answer is that among all the manifestations of “catness” in the world, there is the essence of “cat,” a set of unavoidable properties that something must possess in order to be a cat, and that in fact instantiate all cats.

Unlike its philosophical variant, which consists of a metaphysical thesis, psychological essentialism emphasizes representations of the world, rather than claims about the foundations on which the world is made. It is descriptive rather than normative. Psychological essentialism is less about the claim that things have essences and more about how representations of things of people can reflect this tendency, even if it is wrong (Medin and Ortony, 1989). The main advantage of psychological essentialism is its cognitive frugality. It is a quick and inexpensive heuristic that provides a basis (and a justification) for social categorization (Yzerbyt et al., 1997; Newman and Knobe, 2019). It does not matter whether it is true, as long as it is successful/adaptive under most social circumstances.

Psychological essentialism has been studied from two main perspectives. In the field of education, particularly with regard to the notion of personhood, the study by Carol Dweck on entity vs. incremental mindset has shown the negative consequences which the belief in the non-malleability of self-attributes has on motivation in terms of lack of persistence, low importance attributed to efforts and to learning goals (Dweck, 1999, 2008; Yeager and Dweck, 2012). Social psychologists have focused on social categories, showing that psychological essentialism enhances stereotyping (Bastian and Haslam, 2006), prejudice (e.g., Haslam et al., 2002; Chen and Ratliff, 2018), the acceptance, and the justification of social inequalities (Mandalaywala et al., 2018), and it lessens the desire to enter in contact with racial out-group members (Williams and Eberhardt, 2008). In spite of significant bad press, as noted earlier, psychological essentialism has strategic value for both the individual and the group (Ryazanov and Christenfeld, 2018). Such value has also been discussed in the specific context of fiction by Zunshine (2008) who observed that essentialist thinking not only allows us to place entities into categories but also, and above all, allows us to make inferences (Zunshine, 2008). Without denying their many potential negative consequences, Zunshine (2008) pointed out that such inferences greatly contribute to our ability to understand and deal with the intentions of others. This holds true in both circumstances when we make inferences about natural kinds, for example, other people or a tiger (i.e., when we make inferences about the behaviors of others and behave accordingly) and when we make inferences about artifacts.

Irrespective of its relative value, what is of interest for our purposes is that psychological essentialism is culturally transmitted (Rhodes et al., 2012; Rhodes and Moty, 2020) and remains malleable throughout the lifetime both in terms of mindset (e.g., Levy and Dweck, 1999; Blackwell et al., 2007) and with regard to social categories. Of particular relevance for our hypothesis on the impact of literary fiction on psychological essentialism is a recent study by Pauker et al. (2018), in which white American first-year college students who moved to Hawaii, a racially diverse environment with a high multiracial population, were followed over a 9-month period. They found that the essentialist beliefs of these students about race decreased over time and that this was due to an increase in the ethnic diversity of the acquaintances of students (see also, Young et al., 2013). According to the authors of these studies, exposure to diversity creates uncertainty and challenges category boundaries, hence reducing psychological essentialism. As noted earlier, when the social world is complex and not easy to categorize, essentialism ceases to be a valid heuristic to construe and make sense of it.

Our hypothesis concerning the impact of literary fiction is based on the same premises. Literary fiction exposes readers to the diversity and complexity of humanity, and as such it reduces the extent to which readers engage in psychological essentialism. To test this hypothesis, we relied on the ART as a measure of exposure to literary vs. popular fiction (Kidd and Castano, 2017; Castano et al., 2020) and on the psychological essentialism scale developed by Bastian and Haslam (2006). A two-step procedure was followed. First, in Study 1, we analyzed the data from three different samples in which the ART, one of the psychological essentialism subscales, a measure of political ideology, and the level of education were collected. The latter two variables are important to exclude possible confounds of the ART scores, as detailed in the following section. These samples were collected for different purposes and at different times. The heterogeneity of the samples means that there is considerable noise that might interfere with the relationship under investigation in this study, resulting in a rather conservative test of the same. Second, we conducted a pre-registered study (Study 2), in which the ART, the three subscales of the essentialism scale, a measure of political ideology, and level of education were measured. Other variables were also included in this data collection, but as per the pre-registration, this was done for exploratory purposes and the data are not presented in this study.

## Study 1

Participants (residents of the United States) were recruited *via* Amazon Mechanical Turk (MTurk) and paid from US$2 to US$5, depending on the length of the specific study they took part in. MTurk is a crowdsourcing marketplace for work which is used extensively in behavioral research and has been proven to be a reliable source of good quality data (Crump et al., 2013). Out of a total sample of 795 participants, 37 participants were excluded for failing to select at least one author or selecting more foils than authors on the ART, leaving a sample of 758 (415 females, 1 undeclared; age: *M* = 34, range: 18–82; education: some high school [3], high school [94], some college [271], college graduate [325], and graduate degree [65]).

### Measures

The ART suggested by Acheson et al. (2008) was used. While self-reports of reading habits may be unduly influenced by the desire to appear well-read/knowledgeable, the ART is less likely to suffer from this shortcoming because it includes names of non-authors, which can then be used to assess (and statistically correct for) the tendency of the participants to inflate their knowledge of fiction authors. Also, the ART predicts the actual engagement of the participants with fiction (Stanovich and Cunningham, 1992; Stanovich et al., 1995; Rain and Mar, 2014), and the modified versions have been used in the previous work to compare exposure to non-fiction and fiction (e.g., Mar et al., 2006), and different genres of fiction (e.g., Fong et al., 2013, 2015). Most importantly for present purposes, and as discussed earlier, the ART of Acheson et al. (2008) has been shown to have a two-factor structure that corresponds to exposure to literary and popular fiction authors (Moore and Gordon, 2015; Kidd and Castano, 2017) and that separate scores computed for literary and popular fiction differentially predict social cognition processes and thinking styles (Kidd and Castano, 2017; Castano et al., 2020).

We thus followed exactly the same procedure used in the earlier research (Kidd and Castano, 2017; Castano et al., 2020) to compute ART Literary, ART Popular, and ART Foil scores as proportion of the selection of each category: ART Literary *M* = 0.29, *SD* = 0.23; ART Popular *M* = 0.28, *SD* = 0.23; ART foils *M* = 0.01; *SD* = 0.04. The discreteness subscale of the essentialism scale developed by Bastian and Haslam (2006) was used, which includes eight items (e.g., “People can behave in ways that seem ambiguous, but the central aspects of their character are clear-cut,” “People can have many attributes and are never completely defined by any particular one” (reversed), “Everyone is either a certain type of person or they are not”). An average score was computed (α = 0.85), so that high scores mean a strong tendency to see individuals as falling into discrete categories (*M* = 3.54; *SD* = 0.86). Participants further answered three items asking participants to what extent they self-identified as liberal (1) or conservative (7), in general, from a fiscal and social point of view. A composite score was created by averaging responses to the three items (α = 0.93), with high values indicating more conservative (*M* = 3.33; *SD* = 1.68).

### Results

We used General Linear Model (GLM) (SAS Institute Inc., 2020) with discreteness as the criterion and the ART Literary and ART Popular scores as predictors, and ART Foil, political ideology, and education as covariates. As noted earlier, the data were collected from three different samples. To account for this source of variance, we included Sample as a class covariate. Due to their significant skewness, ART Literary and ART Popular were square-root transformed before being entered in the analysis. The results are shown in Table 1. ART Literary negatively predicted discreteness, while ART Popular did not. Among the covariates, ART Foil was not significant, but Sample was, indicating that discreteness levels varied from one Sample to another. While it is expected, and it does not *per se* impact on the interpretation of the main effects of the ART variables, we conducted further analyses in which the interaction effect between Sample and both ART Literary and ART Popular was included. Neither was significant, and the main effect of ART Literary was unchanged.

**Table 1:** Multiple regression results with discreteness as criterion (Study 1).

**Predictors**	**β**			**95% CI**		**SE**		***F***	***p***	**sr**
ART-Lit	−0.18			−0.30	−0.08	0.05		11.17	<0.001	−0.11
ART-Pop	0.09			−0.01	0.18	0.05		2.78	0.10	0.06
ART-Foil	0.03			−0.04	0.10	0.03		0.90	0.34	0.03
Political ideology	0.16			0.10	0.23	0.04		21.12	<0.001	0.16
Education	−0.02			−0.09	0.05	0.04		0.30	0.58	−0.02
	**Means**			**SDs**						
Sample	3.12	3.57	3.73	0.76	0.88	0.81		23.88	<0.001	0.24

*Model R-square = 0.12*.

## Study 2

Study 2 was pre-registered. Participants (residents of the United States) were recruited *via* MTurk and paid US$1.5 for their participation. Expecting the data loss, we over-recruited (*N* = 300) participants compared with the pre-registered (*N* = 218) participants. After deletion of participants following the pre-registered criteria (*N* = 57), the sample included 243 participants (105 females, 2 undeclared; age: *M* = 40.93; range 18–75). Education was also recorded: elementary school [0]; middle school [0]; some high school [3]; high school [24]; some college [47]; college [121]; and postgraduate [48].

### Measures

The ART was included and scored as for Study 1, and resulted in similar scores: ART Literary *M* = 0.23, *SD* = 0.21; ART Popular *M* = 0.22, *SD* = 0.21; and ART foils *M* = 0.03; *SD* = 0.05. To assess psychological essentialism, in addition to the discreteness subscale (*M* = 3.17; *SD* = 0.70), the two other subscales, namely, informativeness and biological basis, developed by Bastian and Haslam (2006) were used. Informativeness (seven items) assesses beliefs that differences among people allow many inferences to be drawn about them (e.g., “Generally speaking, once you know someone in one or two contexts, it is possible to predict how they will behave in most other contexts”). Biological bases (including eight items) assess beliefs that human attributes are biologically grounded (e.g., “With enough scientific knowledge, the basic qualities that a person has could be traced back to, and explained by, their biological make-up”). A composite score was computed for both constructs, by averaging the items in each scale (informativeness: *M* = 3.45; *SD* = 0.67; biological basis: *M* = 3.31; *SD* = 0.85). Participants also answered one item indicating their self-identification as liberal (1) or conservative (7) with high values indicating more conservative (*M* = 4.16; *SD* = 2.19). They also indicated their level of education on an improved scale comprising seven levels (i.e., elementary school; middle school; some high school; high school; some college; college; and postgraduate).

### Results

We used the same GLM (SAS Institute Inc., 2020) as for Study 1, with ART Literary and ART Popular scores as predictors, and ART Foils, political ideology, and education as covariates, predicting the three subscales of the psychological essentialism scale suggested by Bastian and Haslam (2006), namely, discreteness, informativeness, and biology. As for Study 1, and as pre-registered, variables with considerable skewness were square-root transformed, before being entered in the model. Results (shown in Table 2) replicate the pattern observed for discreteness in Study 1 and further indicate the same effect for the subscale informativeness. Both these effects had been pre-registered. The biology subscale, pre-registered without a strong hypothesis, followed the same pattern.

**Table 2:** Multiple regression results (Study 2).

	**Discreteness**							**Informativeness**							**Biological basis**						
**Predictors**	**β**	**95% CI**		**SE**	***F***	***p***	**sr**	**β**	**95% CI**		**SE**	**F**	***p***	**sr**	**β**	**95% CI**		**SE**	**F**	***p***	**sr**
Lit	−0.24	−0.37	−0.10	0.07	12.01	0.001	−0.20	−0.15	−0.29	−0.01	0.07	4.77	0.030	−0.13	−0.23	−0.40	−0.05	0.09	6.41	0.010	−0.15
Pop	0.04	−0.09	0.16	0.06	0.34	0.560	0.03	0.01	−0.11	0.14	0.06	0.04	0.850	0.01	0.06	−0.10	0.22	0.08	0.51	0.470	0.04
Foil	0.13	0.04	0.22	0.04	8.54	0.004	0.17	0.11	0.02	0.20	0.05	5.77	0.020	0.14	0.11	0.00	0.23	0.06	3.92	0.050	0.12
Political Ideology	0.13	0.04	0.22	0.05	7.93	0.000	0.16	0.07	−0.02	0.17	0.04	2.30	0.130	0.09	0.07	−0.05	0.19	0.06	1.26	0.260	0.06
Education	−0.06	−0.14	0.02	0.04	1.94	0.160	−0.08	−0.04	−0.02	−0.01	0.04	1.07	0.300	−0.06	0.01	−0.09	0.12	0.05	0.06	0.800	−0.04

*Model R-square: discreteness = 0.2102; informativeness = 0.10; biological bases = 0.08*.

In the analysis reported earlier, an unexpected effect of ART foil emerged. The more foils a participant selected as author names, the stronger his/her essentialism score. ART Foil was not transformed because square-root or log transformation did not improve its strongly and positively skewed distribution. Therefore, we conducted further analyses in which instead of using ART Foil as a covariate, we removed it from both ART Literary and ART Popular, to create two “foils-adjusted” versions of both of these variables, and we entered them as a predictor in the same model described earlier. We did this for both Study 1 and Study 2. Results are presented in Tables 3, 4. The predicted effect of (foils-adjusted) ART Literary remains significant in both cases. Interestingly, the (foils-adjusted) ART Popular also emerges as a significant predictor for discreteness, but contrary to ART Literary it positively predicts it. Supplementary Figure 1 shows the residual plots for the original model and the results are shown in Tables 1, 2, and the residual plots for the model presented as supplementary analyses are reported in Tables 3, 4. Supplementary Table 1 reports the bivariate correlations for both Study 1 and Study 2.

**Table 3:** Multiple regression results using discreteness as criterion (Study 1).

**Predictors**	**β**			**95% CI**		**SE**	***F***	***p***	**sr**
Lit-adj	−0.20			−0.31	−0.09	0.06	13.10	<0.001	−0.12
Pop-adj	0.11			0.00	0.21	0.05	4.17	0.04	0.07
Political ideology	0.16			0.09	0.23	0.04	19.94	<0.001	0.15
Education	−0.02			−0.09	0.05	0.04	0.23	0.63	−0.02
	**Means**			**SDs**					
Sample	3.12	3.57	3.73	0.76	0.88	0.81	23.88	<0.001	0.24

*Model R-square = 0.13*.

**Table 4:** Multiple regression results (Study 2).

	**Discreteness**							**Informativeness**							**Biological basis**						
**Predictors**	**β**	**95% CI**		**SE**	***F***	***p***	**sr**	**β**	**95% CI**		**SE**	***F***	***p***	**sr**	**β**	**95% CI**		**SE**	***F***	***p***	**sr**
Lit-adj	−0.34	−0.49	−0.18	0.08	17.63	<0.001	−0.24	−0.21	−0.37	−0.05	0.08	6.34	0.01	−0.15	−0.23	−0.44	−0.02	0.11	4.83	0.03	−0.13
Pop-adj	0.14	−0.01	0.29	0.07	3.55	0.06	0.11	0.05	−0.10	0.20	0.08	0.48	0.49	0.04	0.07	−0.12	0.26	0.10	0.48	0.49	0.04
Political ideology	0.14	0.05	0.23	0.05	8.64	0.00	0.17	0.08	−0.02	0.02	0.05	2.67	0.10	0.10	0.09	−0.03	0.21	0.06	1.96	0.16	0.09
Education	−0.03	−0.11	0.05	0.04	0.59	0.44	−0.03	−0.02	−0.11	0.06	0.04	0.31	0.58	−0.04	0.03	−0.08	0.14	0.05	0.29	0.59	−0.03

*Model R-square: discreteness = 0.21; informativeness = 0.10; biological bases = 0.074*.

## Discussion

In this article, we put forward the hypothesis that exposure to literary, but not popular fiction, is associated with lower psychological essentialism. We tested this hypothesis in two large studies, in one of which (Study 2) we pre-registered the details of data collection and management, as well as the deletion criteria and statistical analyses. The hypothesis found clear support from the results of the analyses of the two studies: exposure to literary, but not popular, fiction was associated with reduced essentialism in terms of perceived discreteness (Study 1) and both discreteness and informativeness (Study 2). The same result emerged on the third subscale suggested by Bastian and Haslam (2006), i.e., biology. The expected effect on this variable was pre-registered as exploratory, but the fact that it emerges and that it parallels that found on the other subscales provides further support for our rationale regarding the effect of exposure to literary fiction on psychological essentialism. To our knowledge, this is the first study of the relation between exposure to literary/popular fiction and psychological essentialism, and thus we cannot compare the strength of the effect we observed in the prior research. We noted, however, that the strength of the relationship between exposure to literary fiction and psychological essentialism is similar to the earlier research findings with regard to the relationship between exposure to literary fiction and ToM (Kidd and Castano, 2017; Castano et al., 2020).

We also conducted supplementary analyses that were not pre-registered, in which a different strategy was adopted for casual or self-aggrandizing responding to the ART—i.e., selection of foils as authors. These analyses revealed the same pattern, i.e., exposure to literary fiction negatively predicted essentialism. They also showed the opposite pattern for exposure to popular fiction, i.e., the more exposure to popular fiction, the greater psychological essentialism.

We first discussed the main hypothesis tested in this study and then discussed the results of the supplementary analyses.

### Significance of the Finding

The main finding confirming our hypothesis adds to the growing literature on the effect of exposure to fiction on social cognition. This work has mostly focused on ToM, but recent findings indicate that exposure to literary fiction is also uniquely associated with attributional complexity for social events, i.e., increased social accuracy and, to a lesser extent, with reduced egocentric bias (Castano et al., 2020). Whether or not psychological essentialism can be considered as inaccurate or leading to biased perception, we perceived a conceptual similarity between these recent findings and those presented in this study: in both cases, literary fiction exposure is associated with a decreased use of reasoning heuristics.

Given the above-mentioned correlates of psychological essentialism, exposure to literary fiction seems to provide the same benefits as those gained through exposure to diversity in the real world (Pauker et al., 2018). Research on the contact hypothesis (Allport, 1954) has shown that entering into contact with actual members of an out-group results in less prejudicial attitudes and stereotyping (Pettigrew and Tropp, 2006, 2008), even when a social contact is mass-mediated (Schiappa et al., 2005). For instance, reduced prejudice and conflict may result from parasocial intergroup contact in virtual environments, i.e., the Internet, TV, movies, and radio (Schiappa et al., 2005; Ramiah and Hewstone, 2013). These studies, however, show context-specific effects, i.e., the stereotype is reduced with regard to the out-group whose members the person enters into contact with. In this study, we suggested that literary fiction undermines one of the very heuristics that supports stereotyping and prejudice, namely, psychological essentialism. The results presented in this study may also thus contribute to the understanding of the dynamics of stereotype change and prejudice reduction through parallel mechanisms to those already highlighted in the social psychological literature.

### Does Exposure to Popular Fiction Matter?

We predicted and found that literary fiction has an inhibiting effect on psychological essentialism. We did not predict that popular fiction would have the opposite effect, and the results of the main analyses suggest that it does not. Why not? If, as it has been suggested (e.g., Kidd and Castano, 2013; Castano et al., 2020), popular fiction reifies social categories, one could expect that greater exposure to it would result in greater psychological essentialism. The reason why we did not predict such a pattern (see pre-registration) is that the kind of category-based perception that is associated with essentialism is likely to be engaged in the absence of a specific motivation to individuate (Ruscher and Fiske, 1990) or in the absence of information that challenges the group stereotype (Rubinstein et al., 2018). In fact, it has also been argued that the individuated appraisal-like process of ToM is not something we engage in with high frequency in our daily life, and that we rather rely on schematic information to navigate and make sense of our social world (see Theory of Society, Hirschfeld, 2006). Furthermore, we would conjecture that psychological essentialism, be it in the form of the essentialization of social categories such as nationality or gender, personality types, or the self, stems from social practices that start early in life, notably through the use of generic language. Gelman and Hirschfeld (1999) wrote that “It seems plausible […] that children learn their essentialist beliefs from the messages directed toward them by mass media (including educational books and TV programs as well as *popular fiction*) and by parents” (p. 423–424; emphasis added). In other words, at least in the Western world, we teach our children to think in essentialistic terms, probably because of the strategic advantage that such thinking provides (Ryazanov and Christenfeld, 2018). Important cultural differences may exist between the Western world and other areas of the world, such as Southeast Asian, where the research has shown that through storybooks, different models of agency are conveyed (Goyal et al., 2019).

While Gelman and Hirschfeld (1999) also noted that powerful counter-essentialist imagery is provided in fiction for children such as *Horton Hears a Who* by Dr. Seuss, it is rather clear that essentialistic mental training is more pervasive. After all, popular fiction is *popular*. Be it with regard to children or an adult audience, narrative fiction that is considered popular is typically perceived as more enjoyable and easier to access, precisely because it can be read using heuristics—which, in turn, are reinforced by its reading. Literary fiction, on the other hand, primes and requires (and thus exercises) a different set of social cognition processes, in which the research shows to be more akin to individuated perception. Literary fiction, therefore, might be undermining, or providing a counterpoint to, the default mode of social perception. For this reason, we believed that while theorizing is consistent with the theoretical perspective proposed in this study, a positive effect of exposure to popular fiction on category-based perception and thus also on essentialism might be difficult to prove empirically. Further research, however, may well find conceptually similar findings, possibly using correlates of psychological essentialism.

Notwithstanding the above, the reanalysis of both studies presented in this study, in which a different strategy to control for the effect of careless or self-aggrandizing responses on the ART was used, revealed, for discreteness, an effect of exposure to popular fiction that is consistent with the rationale concerning the possible effects of popular fiction presented in this study and in other publications (e.g., Kidd and Castano, 2013, 2017; Castano et al., 2020). The alternative analytical strategy that results in this pattern is just as valid as the main one used in this study, and in fact, has been used in earlier work on the ART (e.g., Acheson et al., 2008). Our choice to use the covariate approach, and pre-register it for Study 2, was dictated by the fact that it is the approach used in earlier work which has distinguished, as we did in this study, ART scores for literary and popular fiction (e.g., Kidd and Castano, 2017). Further work on the psychometric properties of the ART will improve our understanding of the implications of using different strategies. In this study, we decided to present both.

### Correlational vs. Experimental Research

Earlier research showing the differential impact of exposure to literary vs. popular fiction on ToM is complemented by experimental work (e.g., Kidd and Castano, 2013). Future research may also provide experimental, rather than correlational, evidence for the relationship between exposure to literary fiction and psychological essentialism reported in this study. Experimental research on the impact of fiction on ToM utilizes, however, performance measures, which we suspected are more sensitive to manipulation in the context of an experiment. The measure of psychological essentialism that we used, just as other measures, is the self-report indications about beliefs, rather than performance measures. The effect of an experimental manipulation on this type of measure might be more difficult to prove empirically, but our rationale, of course, predicts it. The advantage of experimental research, aside from providing a stronger basis for claims about causality, is that it allows for the investigation of mediating factors. An interesting mediating hypothesis to investigate concerns the presence of generic language, which the research has shown to be directly linked to the development of essentialistic thinking in children (Rhodes et al., 2012; Segall et al., 2015; Gelman and Roberts, 2017). It could be that literary fiction makes lesser use of generic language than popular fiction. Other possible mediators are the evaluations provided by participants of the complexity of the characters and especially of their typicality with regard to their main social identities. Although it would not allow to test its mediating effect, analyzing the perception of fictional characters already available on platforms online might provide indirect evidence in support of the idea that category-resistant characters populate literary with greater frequency than popular fiction.

### Limitations

We have already mentioned the limitation stemming from the correlational character of the results presented. In this study, we drew attention to other limitations. One limitation is that evidence from Study 1 has been obtained from a collection of three different samples, rather than an *ad hoc* collected sample of participants. The same sample-aggregation technique was used in the other two articles that factor-analyzed the ART (Moore and Gordon, 2015; Kidd and Castano, 2017). As noted earlier, however, this might also be considered a strength, rather than a weakness—especially in light of the fact that the expected effect emerges while controlling for sample effects. Furthermore, the fact that the pattern was replicated in the pre-registered study using Study 2 is reassuring in this regard. Nonetheless, more data, ideally the cross-cultural data, are needed. The second limitation is the number of covariates we included. Earlier research using the ART, also differentiating literary and popular fiction scores, has ruled out the confounding roles of variables such as personality traits, intelligence, empathic tendencies, or college major (e.g., Mar et al., 2009; Kidd and Castano, 2017; Castano et al., 2020). In this study, we further controlled for political ideology, which is known to be associated with psychological essentialism, and level of education, which may be loosely associated with reading habits. The impact of exposure to literary fiction proved robust to the influence of these variables, but future research may identify and test the impact of other correlates of either exposure to fiction or psychological essentialism, and refine or refute the pattern that we reported in this study.

## Conclusion

In this study, we presented an evidence that exposure to literary fiction is associated with lower psychological essentialism. This finding is consistent with theorizing about the role of fiction in shaping not only *what* we think about the social world, but also *how* we think about it. It complements and extends the emerging body of empirical research on the impact of fiction, both in written form and in other formats, and it further shows the contributions to the debate around the cultural transmission of social cognition processes and thinking styles.

## Data Availability Statement

The raw data supporting the conclusions of this article will be made available by the authors, without undue reservation.

## Ethics Statement

The studies involving human participants were reviewed and approved by Comitato Etico—Dipartimento COSPECS. The patients/participants provided their informed consent to participate in this study.

## Author Contributions

EC developed the hypothesis, designed study, and wrote first draft. MPP, OGC, VC, and PP contributed to preparation of material, data interpretation, and writing. All authors have approved the final version.

## Conflict of Interest

The authors declare that the research was conducted in the absence of any commercial or financial relationships that could be construed as a potential conflict of interest.

## Supplementary Material

Image 1 Figure 1A.

Image 2 Figure 1B.

Image 3 Figure 1C.

Image 4 Figure 1D.
