# Supplementary figures and images for: Exposure to Literary Fiction Is Associated With Lower Psychological Essentialism
Source: Front Psychol. 2021 Jun 8;12:662940. doi: 10.3389/fpsyg.2021.662940 (PMC8217818; doi:10.3389/fpsyg.2021.662940)

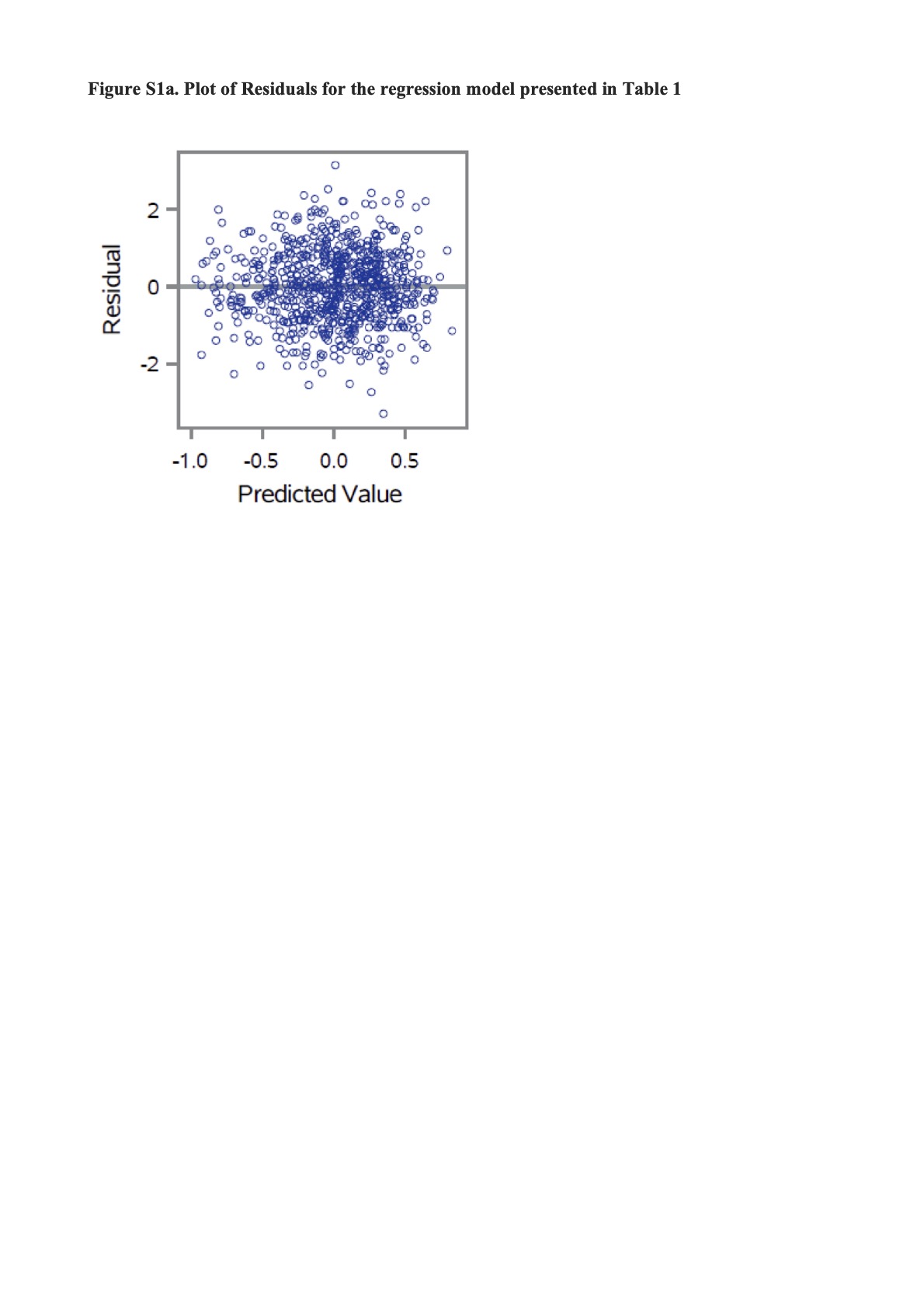

Supplement: Image 1 — Figure 1A. [file Image_1.JPEG]

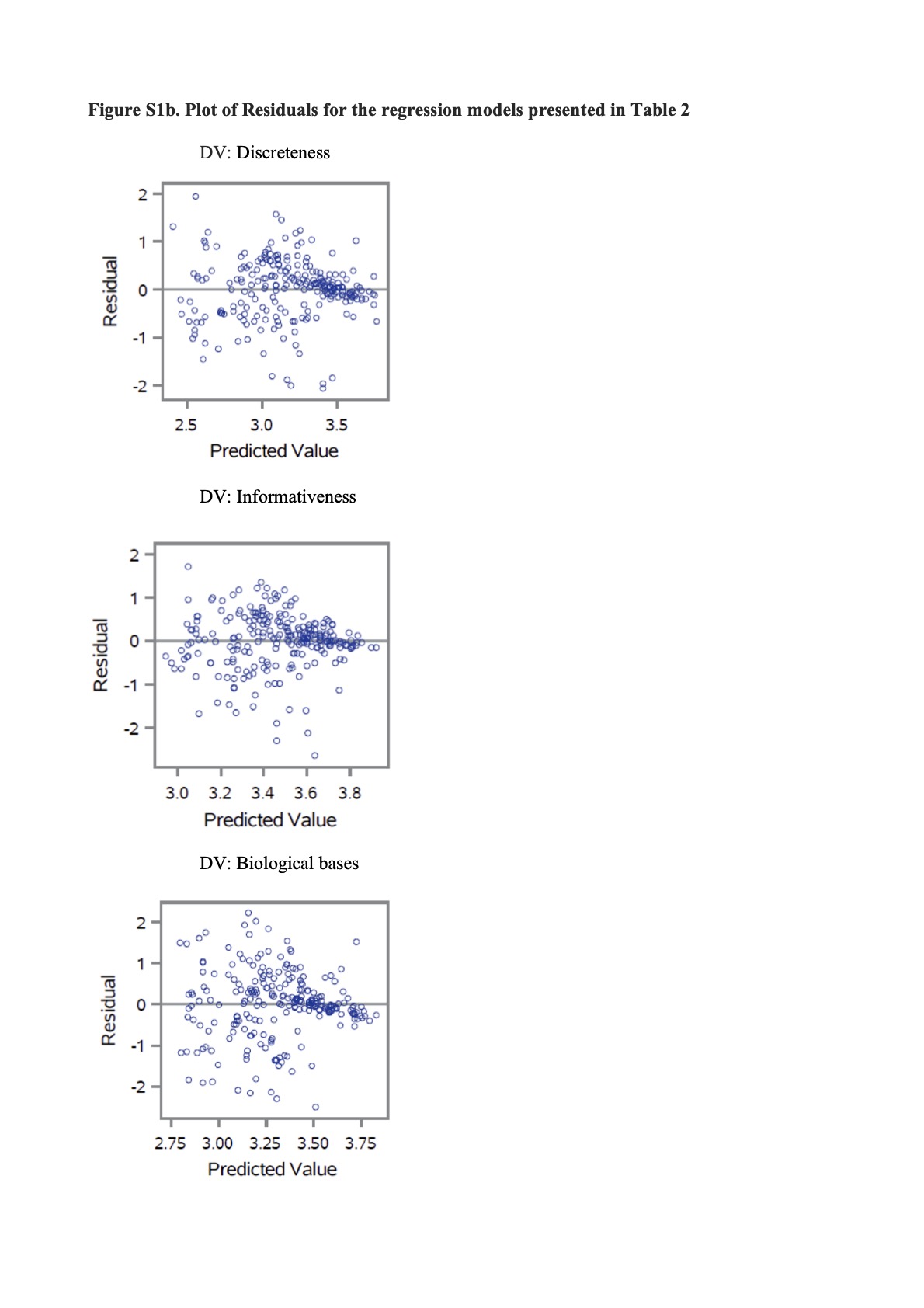

Supplement: Image 2 — Figure 1B. [file Image_2.JPEG]

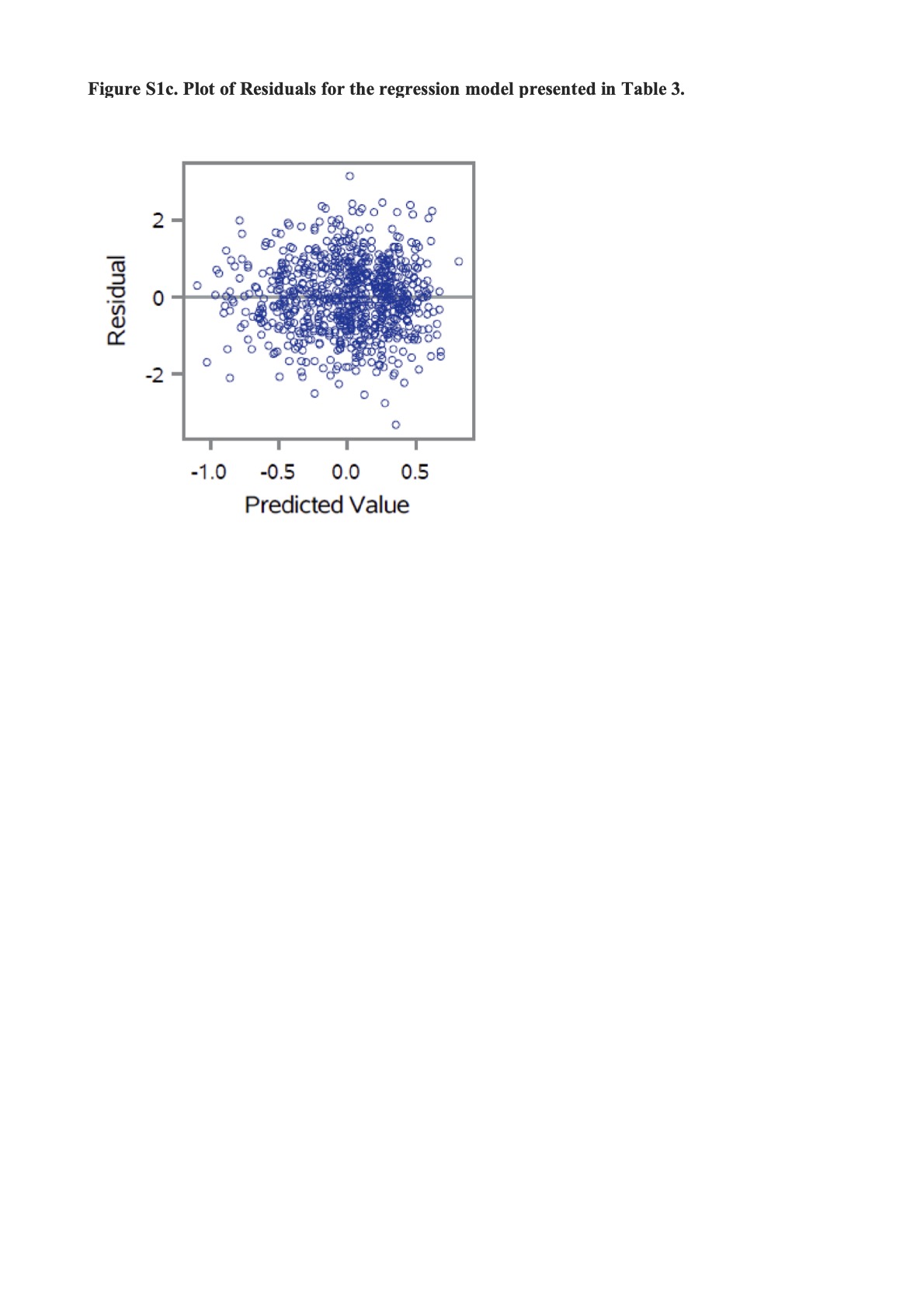

Supplement: Image 3 — Figure 1C. [file Image_3.JPEG]

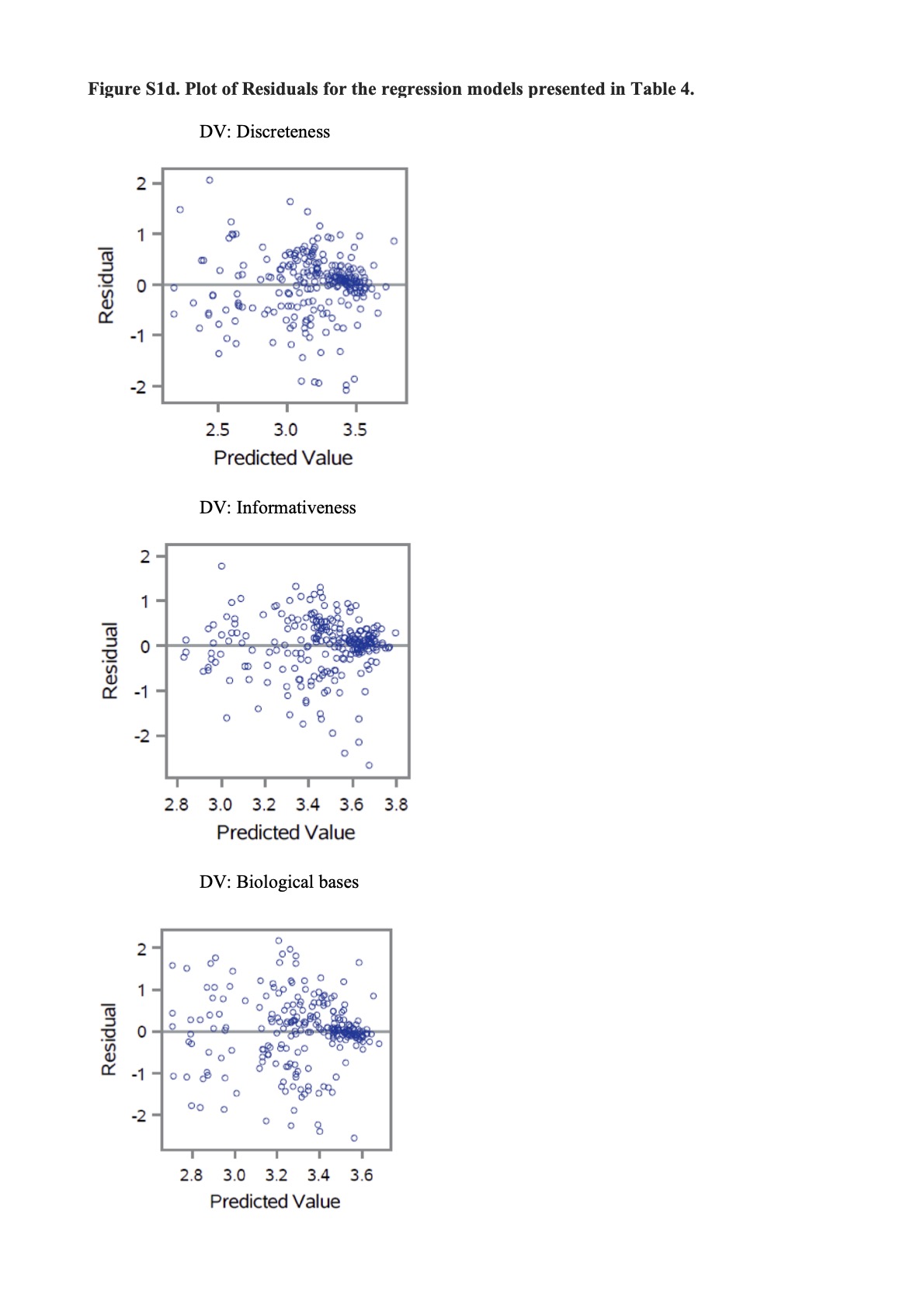

Supplement: Image 4 — Figure 1D. [file Image_4.JPEG]
