# Supplementary material for: Exposure to Literary Fiction Is Associated With Lower Psychological Essentialism
Source: Front Psychol. 2021 Jun 8;12:662940. doi: 10.3389/fpsyg.2021.662940 (PMC8217818; doi:10.3389/fpsyg.2021.662940)
Supplement: Supplementary file 5 [file Table_1.PDF]

Table 1S. Bivariate correlations

	Discreteness	Art-Lit	Art-Pop	Art-Foil	Lit-adj	Pop-adj	Political Ideology	Education	Informativeness	Biological bases
**Discreteness**	--	**-0.19**	*-0.09*	-0.004	**-0.2**	-0.09	**0.2**	*-0.08*	n.a	n.a
**Art-Lit**	**-0.35**	--	**0.74**	**0.19**	**0.95**	**0.72**	**-0.22**	**0.33**	n.a	n.a
**Art-Pop**	**-0.24**	**0.76**	--	**0.18**	**0.72**	**0.93**	-0.08	**0.21**	n.a	n.a
**Art-Foil**	*-0.17*	*0.14*	0.11	--	0.012	0.006	-0.03	0.02	n.a	n.a
**Lit-adj**	**-0.40**	**0.9**	**0.74**	*-0.2*	--	**0.76**	**-0.22**	**0.33**	n.a	n.a
**Pop-adj**	**-0.26**	**0.74**	**0.9**	**-0.20**	**0.83**	--	*-0.08*	**0.21**	n.a	n.a
**Political Ideology**	**0.35**	**-0.39**	**-0.25**	**0.26**	**-0.45**	**-0.30**	--	*-0.09*	n.a	n.a
**Education**	-0.06	*0.123*	0.05	*0.2*	0.091	0.01	*-0.09*	--	n.a	n.a
**Informativeness**	**0.73**	**-0.24**	*-0.17*	*0.15*	**-0.3**	**-0.30**	**0.23**	-0.04	--	n.a
**Biological bases**	**0.56**	**-0.22**	*-0.14*	*0.13*	**-0.25**	*-0.18*	**0.2**	0.02	0.56	--

Above the diagonal, Study 1; below the diagonal, Study 2. Bold $p < .001$; italic $p < .05$
